# Catching's Compendium of Practical Dentistry for 1894

**Published:** 1895-06

**Authors:** 


CATCHING'S COMPENDIUM OF PRACTICAL DENTISTRY FOR 1894.—Since the first number of this work appeared, that for 1880, it has been a welcome annual visitor to the profession. The present number is an improvement upon its predecessors; in fact each year's edition has shown a marked improvement in not only the size but the nature of the contents of this work.

Dr. Catching is to be congratulated for the success of his endeavor to produce such a digest of the contents of the journalistic literature of the dental profession.

B. H. Catching, D. D. S., Editor and Publisher, Atlanta, Ga.

